# Complement Activation as a Helping Hand for Inflammophilic Pathogens and Cancer

**DOI:** 10.3389/fimmu.2018.03125

**Published:** 2019-01-10

**Authors:** Marcin Okrój, Jan Potempa

**Affiliations:** ^1^Department of Medical Biotechnology, Intercollegiate Faculty of Biotechnology, University of Gdańsk and Medical University of Gdańsk, Gdańsk, Poland; ^2^Department of Oral Immunology and Infectious Diseases, University of Louisville School of Dentistry, Louisville, KY, United States; ^3^Faculty of Biochemistry, Biophysics and Biotechnology, Jagiellonian University, Kraków, Poland

**Keywords:** inflamation, periodontits, cancer, *Porphyromonas gingivalis*, complement activation

## Abstract

The complement system, an evolutionarily ancient component of innate immunity, is capable of protecting hosts from invading pathogens, either directly, by lysis of target cells, or indirectly, by mobilization of host immune mechanisms. However, this potentially cytotoxic cascade must be tightly regulated, since improperly controlled complement can damage healthy cells and tissues. The practical importance of this axis is highlighted when impairment of complement regulators or bacterial mechanisms of complement evasion result in pathogenic conditions. Recognition of complement as a “double-edged sword” is widely acknowledged, but another, currently underappreciated aspect of complement function has emerged as an important player in homeostatic balance—the dual outcome of complement-mediated inflammation. In most cases, the proinflammatory properties of complement are beneficial to the host. However, certain pathogens have developed the ability to utilize local inflammation as a source of nutrients and as a way to establish a niche for further colonization. Such a strategy can be illustrated in the example of periodontitis. Interestingly, certain tumors also seem to benefit from complement activation products, which promote a proangiogenic and immunosuppressive microenvironment.

## Introduction

The term “inflammo-*philic*” (= loving or attracting inflammation) was introduced in 2014 by George Hajishengallis to describe dysbiotic microbiome on the tooth surface below the gum line, which thrive in the inflammatory environment of periodontal pockets (1). Remarkably, as described in details later in this review, bacteria responsible for initiation and progression of periodontitis (periodontopathogens) have the unique ability to manipulate the complement system to disengage bacterial clearance from inflammation.

In general, the local inflammatory response to bacterial and fungal pathogens triggered by complement activation is absolutely essential to eliminate invaders (2, 3). Therefore, all successful pathogens developed a large variety of means to interfere with complement activation and/or hinder complement-dependent bacterial clearance mechanisms (2–6) (Table 1). Unfortunately, if inflammatory reaction triggered by pathogens escape the control it becomes highly detrimental to the host as illustrated by invasive candidiasis [*Candida albicans* (7)], meningitis [*Neisseria meningitidis* (8)], and sepsis [*N. meningitidis* (8), *Staphylococcus aureus* (9), and *Streptococcus pyogenes* (10)]. It needs to be kept in mind that an overwhelming inflammatory response and a dysregulated immune response to these infections is by no means the manifestation of an inflammophilic character of these pathogens since the controlled, local inflammation is protective against these pathogens (9). Therefore, the pathogenic strategy to endure inflammation but in the same time to take advantage of it, seems to be limited to periodontopathogens. It is fascinating that the apparently similar strategy is employed by cancer and in both cases exploitation of the complement system underlines pathology.

**Table 1:** Exemplary complement evasion strategies used by microbes.

**Mechanism**	**Organism**	**Protein or molecule/*Host target***
Recruitment of host soluble complement inhibitors: -> support of proteolytic cleavage of C3b and C4b, acceleration of convertases' decay	*Streptococcus pyogenes*	M protein family (b) *Factor H C4BP* FH-binding proteins: fibronectin-binding protein (FbaA) (b) and streptococcal collagen-like protein 1 (Scl1)(b) *Factor H FH-related protein 1*
	*Escherichia coli*	OmpA: Outer membrane protein (b) *C4BP*
	*Moraxella catarrhalisis*	Usp1, 2: Ubiquitous surface protein 1 and 2 (b) *C4BP*
	*Borrelia burgdorferi*	BbCRASP-1 (b) *Factor H*
	*Candida albicans*	Gpm1p (b) *C4BP*
	*Streptococcus pneumoniae*	PspC (b) *Factor H*
Physical barrier preventing Fc receptors on phagocytes to contact complement-derived opsonins on bacteria	*Streptococcus pyogenes*	hyaluronic acid capsule (b)
Blocking of receptor of complement components on immune cells	*Staphylococcus aureus*	Chemotaxis inhibitory protein of *Staphylococcus aureus (CHIPS) (s) C5aR*
Proteolytic inactivation of complement components	*Streptococcus pyogenes*	ScpA (s) *C3a, C5a*
		SpeB (s) *C1 inhibitor, C2, C3, C4, C5a C6, C7, C8, C9*
	*Pseudomonas aeruginosa*	*Pseudomonas* elastase (PaE) (s) *C3*
	*Staphylococcus aureus*	Staphylokinase (s) *C3b*
	*Serratia marcescens*	56kDa protease (s) *C5a*
	*Porphyromonas gingivalis*	*gingipains (s) C3,C4,C5*
	*Tannerella forsythia*	Mirolysin (s) *Mannan binding lectin (MBL), ficolins, C4, C5*
Blocking of classical pathway initiation	*Streptococcus pyogenes*	endopeptidase O (PepO) (s) *IgG-C1q interaction*
		IdeS/Mac-1 (s) *IgG (degradation)*
	*Staphylococcus aureus*	Staphylococcal protein A (SpA) (b) *Ig (binding)*
	*Streptococcus sp. gr. G*	Protein G (b) *Ig (binding)*
Interference/function-blocking of complement components	*Streptococcus pyogenes*	streptococcal inhibitor of complement (SIC) (s) *C5b-7*
		Vitronectin binding proteins (VnBPs) (b) *Vitronectin – C9 polymerization*
	*Staphylococcus aureus*	extracellular fibrinogen-binding protein (Efb) (s) *C3*
		staphylococcal superantigen-like protein 7 (SSL-7) (s) *C5*
		Staphylococcal complement inhibitor (SCIN) (s) *blocking of AP and CP/LP C3 convertases*
	*Borrelia burgdorferi*	CD59-like protein (b) *C8, C9*
Moonlighting proteins	*Streptococcus pyogenes*	GAPDH (b),(s) *binds and sequesters C5a*
	*Filifactor alocis*	Acetylornithine transaminase (FACIN) (b) *binding to C3 and activated C3 in complex with factor B*

*(s), soluble protein/molecule*.

*(b), surface—bound protein/molecule. For original references see the review articles with reference numbers 2–6*.

## Complement System

**Figure 1:**
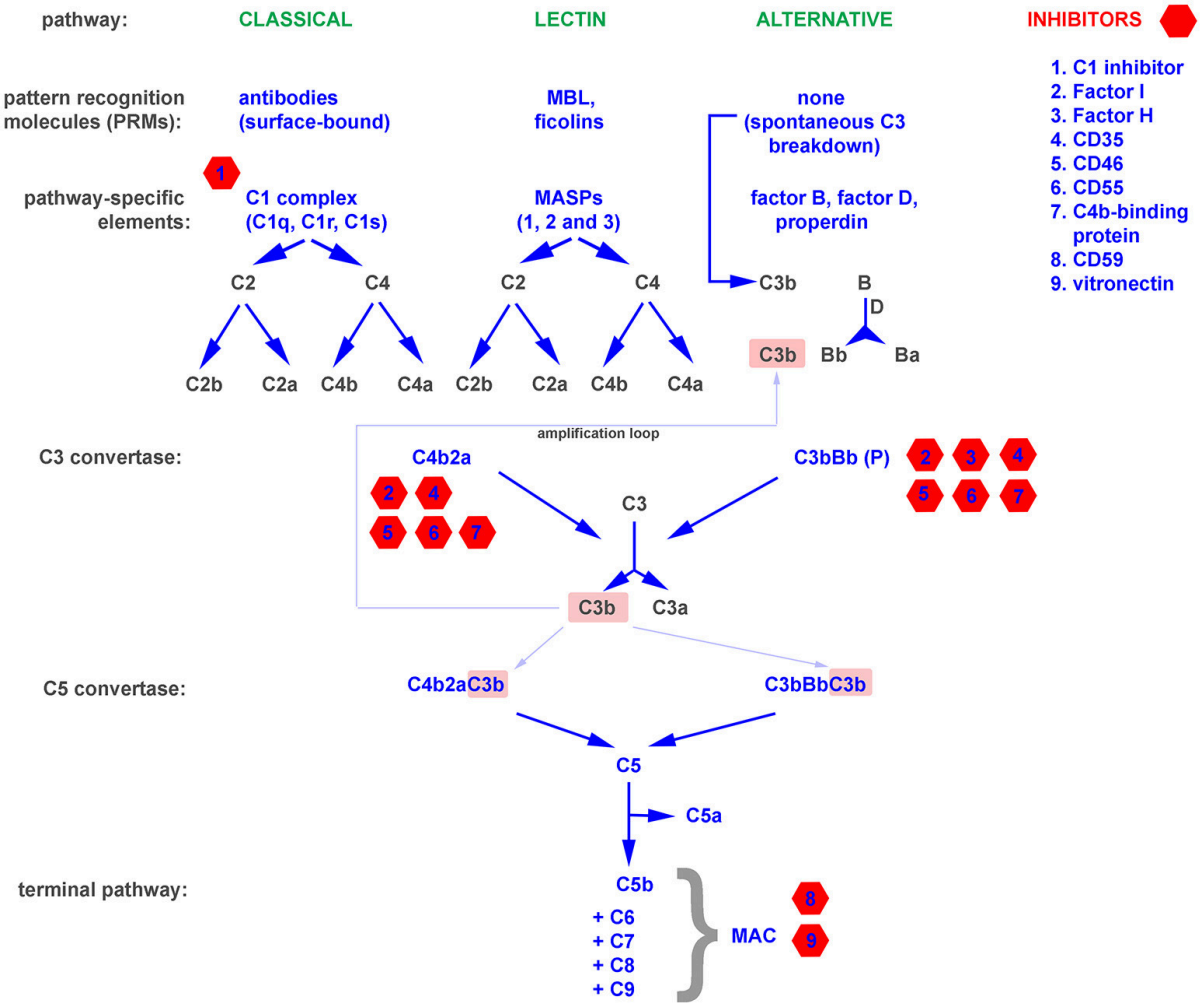
A simplified representation of the complement system, divided into pattern recognition molecules (PRMs), pathway specific and common components as well as inhibitors.

The complement system is one of the oldest mechanisms of immunity. Its essential components, such as the C3 molecule, have existed through more than 500 million years of evolution (11). A primitive complement system probably appeared in the common ancestor of eumetazoa, and its original role was limited to opsonization and the induction of inflammation. Genetic events like the duplication-based appearance of pathway specific components (e.g., factor B and C2) and the gain of terminal pathway constituents (C5–C9) allowed the primodal complement system to evolve into a an advanced and complex defense system capable not only of promoting osmotic lysis of target cells, anaphylaxis, and phagocytosis, but also of crosstalk with other systems (e.g., coagulation) and signaling pathways (e.g., Toll-like receptors) involved in the maintenance of bodily homeostasis (12, 13). There are three independent complement cascades, the evolutionarily older alternative and lectin pathway (with basic elements like C3, MASPs, and factor B existing in invertebrates) and the relatively younger classical pathway developed in jawed vertebrates (11). The alternative complement pathway is constitutively active at a low level due to the spontaneous breakdown of C3 into anaphylatoxin C3a and the active C3b fragment, which activate downstream steps in the cascade. Therefore, propagation of the alternative pathway does not depend on specific activation but relies on the lack of inhibition by numerous endogenous regulators that differentiate self and non-self surfaces. This mechanism ensures constant monitoring of the body. In contrast to the alternative pathway, the classical and lectin pathways require specific stimuli, such as antibodies, C-reactive protein, phosphatydylserine, or certain sugar moieties, to be present on the surface of target cells (14–16). The upstream components of both pathways, including C1q, mannan binding protein (MBL), and the ficolins, act as sensors and thus can be considered soluble pattern recognition molecules (PRMs) (17, 18). All pathways converge at the level of the central complement molecule C3. C3 is processed by enzymatic complexes called complement convertases into C3a anaphylatoxin and the C3b fragment, which in turn forms C5 convertases. C5 convertases cleave the C5 molecule into C5a anaphylatoxin and the C5b fragment, which initiates the common terminal pathway. Binding of C6, C7, C8, and C9 leads to formation of the membrane attack complex (MAC), which targets the cell membrane and causes osmotic lysis. A schematic representation of the complement system is shown in Figure 1.

The binding of antibodies as a stimulus for the initiation of the complement cascade bridges the innate and adaptive immune systems. Moreover, opsonins like C3b and their degradation products act as natural adjuvants, contributing to proper presentation of antigens to lymphocytes and providing a co-stimulatory and anti-apoptotic signal for B cells (19). Deficiencies of complement, while relatively rare, emphasize the importance of this multifunctional protein cascade (20). The exact symptoms that develop depend on precisely which complement components are lacking or impaired. Deficiencies in essential components of the alternative pathway and the terminal pathway result in higher susceptibility to recurrent bacterial infections, especially these caused by Neisseriae and incidence peak up in early childhood (20). The lack of early components of the classical pathway predisposes to systemic lupus erythematosus (SLE), a disease in which the scavenging function of complement is impaired, and thus debris from dying cells persists and can act as a source of autoantigens (21). Autoimmune diseases that stem from direct damage of cells and tissues typically arise from deficiencies in complement inhibitors that normally protect the host from excessive or misguided complement attacks (22). These include C3 glomerulopathies, atypical hemolytic uremic syndrome (aHUS), age-related macular degeneration (AMD), paroxysmal nocturnal hemoglobinuria (PHN), and many more (20). Paradoxically, a deficiency of functional complement inhibitors such as factor H (the main soluble inhibitor of the alternative pathway) can also result in a deficiency of complement activation. Factor H is the main soluble inhibitor of alternative pathway, which prevents propagation of cascade beyond the spontaneous breakdown of C3 and formation of C3 convertase (Figure 1). The lack of such inhibitor fuels a positive feedback mechanism that unproductively depletes complement and leaves the host without an important line of defense (23). On the other hand, unwanted complement activation is an effector mechanism in many inflammatory diseases, including rheumatoid arthritis (24), diabetic nephropathy (25), and ischemia/reperfusion injury (26). All these examples support a perception of the complement system as a “double-edged sword,” where a proper balance is pivotal for maintaining protection while avoiding autoimmunity. Both microbial infections and tumors influence this physiological equilibrium and employ two main strategies for survival in a complement-saturated microenvironment.

## Strategy #1: to Counteract Complement

Innate immunity relies on recognition of a spectrum of pathogen-associated molecular patterns (PAMPs), invariable molecular determinants typical for the most common invaders, including lipopolysaccharide (LPS), lipoteichoic acid, flagellin, double-stranded RNA, β1-3 glucan, N-formylmethionine peptides, and many more. The constant region of the antibody heavy chain (Fc) also falls into this category of molecules. Pathogen-associated molecular patterns (PAMPs) bind to specific receptors on innate immune cells and activate effector mechanisms such as complement or antibody-dependent cell cytotoxicity (ADCC). Molecules that sense PAMPs and trigger immune system activation are called pattern recognition receptors (PRRs) and include Toll-like receptors (TLRs), C-type lectins, and NOD-like receptors. Soluble molecules, which exert an analogical role are termed as PRMs and the most upstream components of the classical and lectin pathways (C1 complex, MBL, and ficolins) belong to this group (18). Complement is therefore a multispecific and powerful defense system against pathogens that is theoretically capable of eliminating every cell unless constrained by endogenous complement inhibitors. In practice, since complement co-evolved with pathogens over millions of years, pathogens have developed various mechanisms to evade complement attack. Pathogens employ a variety of tactics for this purpose, including proteolytic cleavage of complement components, mimicking and hijacking host complement inhibitors, inactivation of the C3 molecule, preventing of complement-mediated activation of immune cells, depletion of antibodies, and unproductive exhaustion of early complement components [reviewed in (2, 4, 27–29)]. Selected examples of abovementioned strategies are given in Table 1.

Similarly to bacterial, fungal, or viral pathogens expressing PAMPs, tumor cells are visible to the immune system due to changes in their mutational or metabolic status, which is reflected by changes in the expression of cell surface molecules. The presentation of epitopes derived from mutated proteins (so-called neoantigens) within MHC I molecules (30) as well as the peroxidation of membrane lipids or changed patterns of glycosylation distinguish tumor cells from normal cells.

Spontaneous fixation of complement onto the surface of tumor cells is of low physiological relevance due to the low titer of naturally occurring antitumor antibodies and the expression of complement inhibitors by tumor cells (31). The introduction of antitumor monoclonal antibodies, which is considered a breakthrough in tumor immunology, enabled researchers to use the cytotoxic potential of the complement system to combat cancer (32). Complement-activating therapeutics like the anti-CD20 antibodies rituximab and ofatumumab are first-line therapies in the treatment of B cell malignancies. However, certain patients fail to respond or only partially respond to antitumor antibodies, and one possible explanation is the unfavorable ratio of the molecular target (e.g., CD20) to membrane-bound complement inhibitors on the surface of tumor cells (33, 34). Successful experiments in which bispecific antibodies against CD20 and CD55 were used (35) or in which complement inhibitors were silenced (36) support the theory that inhibition of complement by tumor cells is an important mechanism of cancer resistance. Expression of membrane-bound complement inhibitors like CD35 (Complement receptor 1, CR1), CD46, CD55, and CD59 is typical for nucleated cells, and the majority of cell types express at least one of these molecules. In contrast, the production of soluble complement inhibitors such as factor I, factor H, C4b-binding protein (C4BP) is usually the domain of liver hepatocytes, and there are only few extrahepatic sources of fluid-phase complement regulators (37). However, the expression of soluble complement inhibitors by tumor cells has been described, and it seems to provide an additional level of protection, as shown in an *in vitro* model of non-small lung cancer cell (NSCLC) lines expressing factor I, C4BP, and factor H (38). The tumor-supporting effect of endogenous factor H expressed by NSCLC cells was shown *in vivo* in a mouse xenograft model (39, 40). Further evidence for the pro-tumor effect of soluble complement inhibitors comes from analysis of tissue microarrays of breast cancer specimens. Expression of factor I was positively correlated with tumor size, de-differentiation score (Nottingham scale), and poor prognosis (cancer-specific survival and recurrence-free survival) (41). Other investigators reported a correlation between factor I expression and tumor aggressiveness in cutaneous squamous cell carcinoma (42). In addition to expressing soluble complement inhibitors, tumor cells can also hijack these proteins from the plasma. Horl et al. showed that blocking factor H binding to the surface of leukemia cells increased the cytotoxicity of rituximab (43) and ofatumumab (44). Although factor H is an inhibitor of the alternative complement pathway, it plays a role in enhancing the complement cascade when it is initiated via the classical pathway (such as by antitumor antibodies) at the level of C3b formation. C3b gives rise to an amplification loop (Figure 1) due to the formation of alternative convertases, which are targets for factor H. Points of action of particular complement inhibitors are indicated in Figure 1. Another possible way to increase tumor cell resistance to complement attack is removal of the MAC from the surface, a process dependent on endocytosis or active rearrangement of the cell membrane mediated by phosphorylation of essential signaling proteins (45).

The logical consequence of complement inhibition by microbes and tumor cells at various stages of the cascade is a more aggressive and more drug-resistant phenotype, as discussed above. However, the picture is not as simple as it may originally seem, and another strategy used by pathogens and tumor cells to evade complement has been described.

## Strategy #2: to Exploit the Complement System

Low oxygen concentration is a feature of rapidly growing solid tumors, which cannot develop the vasculature necessary for the efficient supply of nutrients to proliferating neoplastic cells. Therefore, the expanding tumor mass sooner or later develops hypoxic cores. Normal cells are equipped with a sensor of oxygen concentration that works at the transcriptional level. Hypoxia-inducible factor Iα (HIF-1α) can stabilize the p53 tumor suppressor, triggering either apoptotic signaling or metabolic reprogramming of the cell (46). Both of these processes lead to changes in the molecules expressed at the cell surface, which has the effect of making the tumor cell visible to the immune system. Previous studies with human umbilical vein endothelial cells (HUVECs) revealed that these cells activate the classical complement pathway in response to hypoxia and as well as during subsequent reoxygenation. At the same time, HUVECs increased their surface expression of two membrane-bound complement inhibitors, CD46 and CD55 (47), which induce the proteolytic cleavage of activated complement components C3b and C4b, respectively, and the dissociation of the corresponding complement convertases. Another study showed a 3.6-fold increase in HUVEC expression of complement receptor 1 (CR1 or CD35) after 48 h of hypoxia (48). These results suggest that endothelial cells actively counteract complement activation under hypoxic conditions, and therefore the expression of complement inhibitors in hypoxic NSCLC cells was studied (49). These cells not only expressed membrane-bound complement inhibitors but also produced soluble inhibitors of complement, including C4BP and factors I and H (38). In contrast to HUVECs, NSCLCs significantly downregulated the mRNA expression of all complement inhibitors tested except CD59 after 24 h of hypoxia, but a drop in the mRNA expression of soluble complement inhibitors was detected as early as 6 h after hypoxic challenge. Importantly, this rapid decrease did not correspond to the number of dying cells, which did not significantly increase in first 24 h (49). The conclusion is that unlike endothelial cells, NSCLCs do not utilize protection mechanisms that prevent the deposition of early complement components during hypoxia, but they do maintain expression of CD59, which protects from the terminal stages of complement attack (the insertion of the MAC into the membrane) (47–49).

From the research reviewed above, it has become apparent that lung cancer cells may benefit from the propagation of local inflammation mediated by C3a and C5a. Possible scenarios include the production of proangiogenic and growth factors by tumor-infiltrating lymphocytes and macrophages as well as the mobilization of immune suppressor cells that impair tumor antigen presentation (50–53). Indirect support for this hypothesis comes from studies done by Ajona et al. who reported elevated C4d deposition in lung tumors and its correlation with decreased survival (54). Moreover, high levels of soluble C4d in the plasma could discriminate between patients with benign pulmonary nodules and lung cancer (55), and were associated with reduced survival of individuals with early and advanced lung cancer. C4d levels in the plasma were also reduced after surgical removal of the tumor (54). C4d is an end degradation product of the activated C4b molecule, a hallmark of classical complement pathway activation. For that reason, one can assume that the survival and malignant potential of NSCLC cells is based on stimulation of complement. Indeed, anaphylatoxin C5a is one of the key players in complement-mediated support of lung cancer growth. Corrales et al. found that C5 deposition and subsequent C5a generation in NSCLC cells was much higher than in non-malignant bronchial epithelial cells in the presence of serum (56). Interestingly, tumor cells but not non-transformed cells produced endogenous C5, and C5a generation took place even in the absence of serum. C5a levels in the plasma of lung cancer patients were also found to be elevated, similarly to C4d levels. C5a also stimulated migration and tube formation by HUVECs *in vitro*. Finally, the impact of C5a was tested in a syngenic mouse model of 3LL lung cancer. Microvessel density was compared in 3LL tumors in mice treated with a C5a receptor (C5aR) antagonist. Tumors in the mice treated with the C5aR antagonist showed significantly fewer microvessels (56). Additionally, C5a signaling positively influenced the recruitment of myeloid-derived suppressor cells (MDSCs; CD11b^+^, Ly6c^+^), as blockade of C5aR reduced the number of MDSCs in tumor-bearing mice. The authors also found decreased expression of molecules associated with an immunosuppressive state and silencing of the immune response (ARG1, CTLA-4, IL-10, LAG3, and PD-L1) in C5aR antagonist-treated mice (57–59).

Importantly, the first evidence for the impact of C5a on the mobilization of MDSCs into the tumor mass was shown by Markiewski et al. in the TC-1 tumor model, a lung epithelial cell line expressing human papilloma virus (HPV) E6 and E7 antigens (60). The authors found that C5aR-deficient mice developed smaller tumors than wild-type littermates, and the same effect was observed when a C5aR antagonist was administered. However, in this model, the slower rate of tumor growth in C5aR antagonist-treated animals was not dependent on tumor cell proliferation/apoptosis or angiogenesis, as evidenced by analysis of end-point tumor specimens. Conversely, there were differences in the infiltration of tumor tissue by cytotoxic T cells, the main effectors of the antitumor immune response. Profiling of MDSCs isolated from tumors and spleens of C5aR-deficient, tumor-inoculated animals confirmed that C5a contributes to the accumulation of MDSCs in peripheral lymphoid organs and their migration into tumors. Of note, MDSCs isolated from mice with disabled C5aR signaling were less able to suppress T cell proliferation *in vitro*. This deficiency was linked to lower production of reactive oxygen species (ROS) and reactive nitrogen species (RNS) in mononuclear MDSC from C5aR-deficient animals.

Similarly to lung cancer cells, endogenous C5a generation by pancreatic and colon cancer cells was later reported. These cells processed C5 with a cell surface-expressed serine protease and expressed C5aR, suggesting autocrine activation of complement (61). In ovarian cancer, endogenous production of complement components and autocrine stimulation of the anaphylatoxin receptors C3aR and C5aR was suggested to be an important mechanism supporting tumor growth (62). The observed effect was independent of infiltration by cytotoxic T cells, since experiments with silenced expression of C3 yielded the same result (i.e., reduced tumor growth) in CD8 T cell-sufficient and -deficient mice. A direct effect of C3aR and C5aR agonists on proliferation, migration, and invasion of tumor cells has also been reported. Finally, quantification of C3 mRNA in tumors from patients with ovarian cancer showed that overall survival in patients with low tumor expression of C3 was more than double that of patients with high expression of C3 in the tumor (62).

In recent years, there has also been growing evidence for the pro-tumor activity of anaphylatoxins and anaphylatoxin receptors in either tumor cells or the tumor stroma in multiple tumors types, including melanoma, breast, ovarian, cervical, colon, and intestinal cancer, as well as sarcoma [reviewed in (63)]. Interestingly, in addition to the larger body of work focusing on the role of C3a and C5a in promoting tumor growth, recent studies have described a pro-tumor effect of factor B silencing (64) as well as a complement-independent enhancement of tumor growth, adhesion, and angiogenesis by C1q produced by the tumor stroma (65). The concept of complement activation supporting tumor growth provided the rationale for combined inhibition of C5a and PD-1 (66, 67), suggesting that targeting complement may be an effective anticancer treatment. These novel discoveries may be perceived as contradictory to the acknowledged theory that complement inhibits tumor growth. For example, NSCLC cells, which have been shown to benefit from C5a generation (56), were previously shown to form smaller tumors in a mouse xenograft model when their endogenous expression of the complement inhibitor factor H was silenced (40). In addition, potent complement activators such as the anti-CD20 immunotherapeutics rituximab and ofatumumab are first-line therapies for treatment of B cell malignancies (33). Notably, solid and circulating tumors have different requirements for growth. While sold tumors are typically depend on angiogenesis, migration, degradation of extracellular matrix, liquid tumors originate in the bone marrow, peripheral blood, or lymph nodes, which are rich in both nutrients and complement. Even solid tumors of the same origin can differ one from another in their mutational status, basal expression of growth factors and metalloproteinases, and metabolic rate. All of these parameters can influence the overall effect of complement activation on tumorigenesis and/or tumor progression. Finally, tumor cells often produce both complement activators and complement inhibitors. Thus, it seems as though tumor cells actively regulate the complement system depending on microenvironmental conditions, rather than simply avoiding constitutive inhibition or activation of complement (68).

Despite the extremely long phylogenetic distance between eukaryotic cells and bacteria, some prokaryotes have acquired strategies similar to tumor cells, which utilize the host inflammatory status to create favorable survival conditions. Bacterial growth in the human body is less dependent on neovascularization than tumor growth, and in contrast to tumor cells, bacteria do not have to overcome internal mechanisms controlling proliferation. Moreover, most bacteria can stand much harsher conditions than eukaryotic cells in terms of pH, oxygen tension, temperature, concentration of metabolites, *etc*. Nevertheless, the common feature between bacteria and tumor cells is the demand for nutrients and certain microelements. While solid tumors induce angiogenesis to acquire a source of nutrients, bacteria can successfully utilize products from the breakdown of local tissue. Therefore, tissue-destructive processes linked to local inflammation form permissive conditions for prokaryotic pathogens, which can survive immune attack. An additional benefit of this strategy is the elimination of inflammation-sensitive bacterial species (human commensals or normal microflora) that normally compete within the same niche (69, 70).

## Inflammophilic Character of Porphyromonas Gingivalis, Which Propels Periodontisis

One of the most well-documented examples of bacteria hijacking host immunity to create an environmental niche occurs in periodontitis, a chronic inflammatory disease characterized by dysbiosis that results in degradation of the gingiva and tooth-supporting bone and ultimately leads to tooth loss (1). Periodontal disease begins from dental plaque, a microbial matrix colonizing the gum line usually as a result of inefficient oral hygiene (71). The next stage, gingivitis, is characterized by local inflammatory response to microbial plaque. The switch between non-destructive gingivitis and destructive periodontitis involve dysbiosis of the normal oral microbiome. The dysbiotic process results from an imbalance of homeostasis caused by so-called keystone pathogens (72). A keystone pathogen is usually a microorganism of low abundance that induces changes in the composition of the local microflora by introducing a new selective pressure, such as inflammation. In periodontitis, the keystone pathogen is *Porphyromonas gingivalis*. However, as shown by studies in mice, this Gram-negative bacteria cannot establish periodontitis by itself, but requires commensal microbes. These microbes are then converted from a symbiotic into a dysbiotic community. Pivotal experiments showed that bone loss was reduced when C3aR- or C5aR-deficient mice were inoculated with *P. gingivalis* and that no changes in the oral microbiota were observed in these knockout mice after *P. gingivalis* inoculation, in contrast to wild-type mice (69). As some tumor cells generate C5a through their surface enzymes, so does *P. gingivalis*. It is equipped with gingipains, outer membrane-anchored bacterial surface arginine-specific proteases with C5 convertase-like activity (73, 74). Importantly, gingipains release C5a from C5, but at higher concentrations, they degrade the larger fragment (C5b), thus preventing MAC formation (75). C3 and C4 complement proteins are also degraded by high concentrations of gingipains. Thus, human serum pre-incubated with clinical strains of *P. gingivalis* but not mutants lacking gingipains is devoid of bactericidal activity (74, 76). Additionally, gingipains interact with the C1 complex and increase its deposition onto bacteria surfaces (74). Based on these findings, one can postulate a biphasic effect of *P. gingivalis* proteolytic enzymes. A low abundance of bacteria initiates the classical complement pathway, but increasing numbers of bacteria results in the degradation of crucial complement components, leading to osmotic lysis. C5 is present in gingival crevicular fluid at concentration corresponding to 70% of that in serum and the active C5a anaphylatoxin can be locally released by convertases and bacterial proteases (77). C5a is a strong inflammatory mediator that increases vascular permeability and attracts and modulates the function of neutrophils, monocytes, and mast cells. All these events are considered antimicrobial events. Paradoxically, *P. gingivalis*' strategy for immune subversion by proinflammatory C5a involves targeted immunosuppression of macrophages. C5a affects intracellular killing of engulfed *P. gingivalis* by RNS and corrupts the crosstalk between C5aR and TLR2, one of the most important PRMs in antibacterial innate immunity (73). At the same time, C5aR-TLR2 crosstalk results in release of proinflammatory cytokines such as IL-1β, IL-6, and TNF-α, which accelerate bone resorption and thus contributes to the pathological mechanism of periodontitis. Similarly, *P. gingivalis* spoils intracellular killing mechanism but not proinflammatory activity of neutrophils by degradation of TLR2 adaptor molecule MyD88 provoked by concomitant activation of TLR2 and C5aR (78).

Another functional similarity between *P. gingivalis* and tumor cells, which produce either complement inhibitors or complement components, is the fact that *P. gingivalis* not only possesses the proteolytic machinery to generate C5a but also expresses a unique enzyme, peptidyl arginine deiminase (PPAD), which can citrullinate the C-terminal arginine in C5a, a modification that results in substantial loss of anaphylatoxin chemotactic activity (79). This suggests that the evolutionary goal of pathogens like *P. gingivalis* is not constitutive activation or inhibition of the complement system, but rather the ability to actively control complement status depending on its current needs. As a keystone pathogen, *P. gingivalis* is a low-abundance species that plays a major role in remodeling the local microbiota community (69). Following establishment of *P. gingivalis* infection, a succession of other dysbiotic species proliferates in the periodontal plaque. Some express their own complement inhibitors, such as *Tannerella forsythia*, which produces karilysin and mirolysin (80, 81), *Filifactor alocis*, which produces FACIN (82), and *Prevotella intermedia*, which produces interpain A (83). Of note, interpain A works in concert with gingipains in the initial stages of infection, as both proteins activate the C1 complex and increase its deposition onto the cell surface. A dysbiotic bacterial community may promote a transcriptomic response that further improves bacterial fitness by regulation of nutrient acquisition and expression of virulence factors (84). This process resembles the crosstalk between tumor cells and the stroma, at least to a certain extent. Tumor cells can drive the polarization of infiltrating immune cells (e.g., into M2 macrophages), which in turn benefit the tumor cells by, for example, expressing angiogenic cytokines (85, 86). In addition, carcinoma-associated fibroblasts (CAF), which differentiate from normal fibroblasts upon stimulation by cancer-derived cytokines such as TGF-β, have emerged as important players in cancer progression and metastasis (87, 88).

## Concluding Remarks

**Figure 2:**
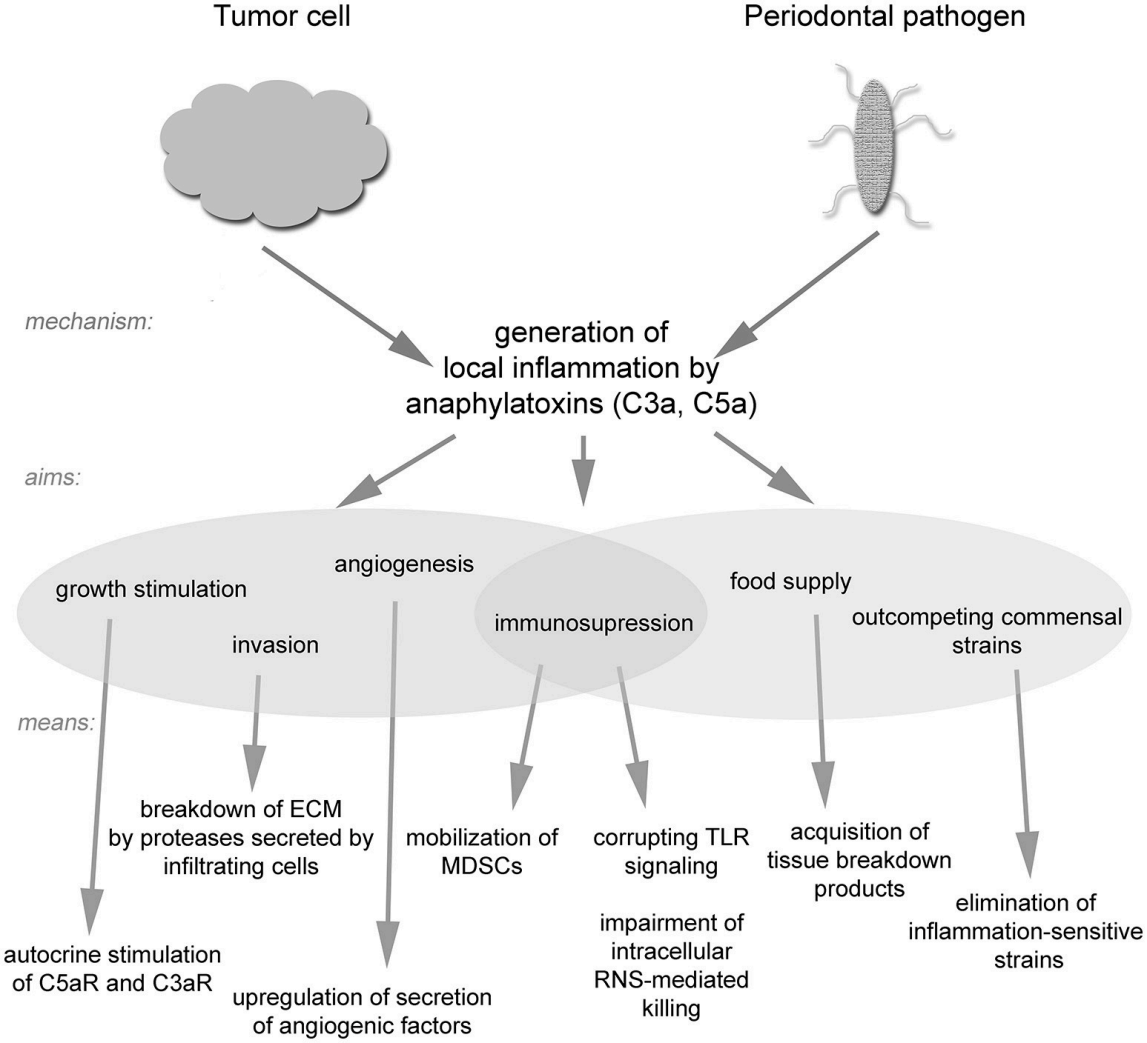
A schematic drawing of strategies utilized by tumor cells and inflammophilic bacteria to subvert complement activation.

The role of the complement system in combating bacteria and cancer is more complicated than was initially believed. Certain pathogens have evolved the ability not only to evade complement attack but also to use it as a tool for establishing their own niche, while remaining protected from complement-mediated lysis. Such a strategy seems to be widespread in nature and has been adopted by both bacteria and tumor cells (Figure 2). It seems likely that additional pathogenic strategies remain to be discovered, and thus one must be careful when designing complement-based therapeutics. On the other hand, anti-complement approaches may be effective in the treatment of infections caused by inflammophilic microbes.

## Author Contributions

All authors listed have made a substantial, direct and intellectual contribution to the work, and approved it for publication.

### Conflict of Interest Statement

The authors declare that the research was conducted in the absence of any commercial or financial relationships that could be construed as a potential conflict of interest.
